# Temperance in Congress

**Published:** 1867-09

**Authors:** 


TEMPERANCE IN CONGRESS.—"Ten-Minute Speeches" by Hon. Schuyler Colfax, Henry Wilson, Richard Yates, Wm. E. Dodge, Hiram Price, Samuel McKee, F. E. Woodbridge, J. B. Grinnell and J. W. Patterson, delivered at the first meeting of the Congressional Temperance Society, Washington, D. C. With a list of Pledged Members. New York: S. R. Wells, Publisher, 389 Broadway. Price 25 cents.

"TEN-MINUTE SPEECHES," or "Temperance in Congress," is beautifully printed on tinted paper, in large clear letter, and is every way worthy of the great occasion which called it forth. We would have a copy placed in the hands of every young man in the nation. It would serve to fortify him in resisting temptations, which will sooner or later beset him. Walled in with the best resolutions, he is still liable to fall. This concentrated and powerful appeal will help such a one to keep his resolutions, and MUST produce conviction in the heart of the skeptic, and HOLD the convert.

Reader, place a copy of "Temperance in Congress" in the hands of the one you love best.

